# What Are the Opportunities and Challenges of Using AI in Medical Education in Vietnam?

**DOI:** 10.2196/77817

**Published:** 2025-12-02

**Authors:** Trung Anh Nguyen, Thanh Binh Nguyen, Duy Cuong Nguyen, Anh Dung Vu, Khanh Linh Dang, Nhu Quynh Le, Duy Anh Ngo, Dang Kien Nguyen, Van Thuan Hoang, Thanh Binh Ngo

**Affiliations:** 1 Thai Binh University of Medicine and Pharmacy, 373 Ly Bon Street, Hung Yen, 06000, Vietnam, 84 2273838545, 84 2273847509; 2 Hanoi University of Science and Technology, Ha Noi, Vietnam

**Keywords:** artificial intelligence, medical education, Vietnam, digital transformation, adaptive learning, virtual simulation, curriculum innovation, educational technology

## Abstract

Artificial intelligence (AI) has the potential to transform medical training through adaptive learning, immersive simulations, automated assessments, and data-driven insights, offering solutions to persistent issues such as high student-to-faculty ratios, overcrowded classrooms, and limited clinical exposure. Globally, many universities have already embedded AI literacy and competencies into undergraduate, postgraduate, and continuing education programs, while in Vietnam, the use of AI in medical education remains limited and fragmented. Most students have little formal exposure to AI, and empirical evidence on faculty or institutional readiness is scarce. Experiences from other countries, including Malaysia, Palestine, and Oman, demonstrate that incremental adoption and faculty development can facilitate cultural acceptance and curricular innovation, providing useful lessons for Vietnam. At the same time, significant barriers remain. These include inadequate infrastructure in provincial universities, low levels of AI literacy among both students and educators, underdeveloped regulatory and ethical frameworks, and resistance to pedagogical change. Cost-effectiveness and sustainability are additional concerns in a middle-income context, where upfront investments must be balanced against long-term benefits and equitable access. Advancing AI in Vietnamese medical education will therefore require a coordinated national strategy that prioritizes infrastructure, AI literacy, faculty development, quality assurance, and sustainable funding models, alongside ethical and legal safeguards. By addressing these key foundations, Vietnam can harness AI not only to modernize medical education but also to strengthen preparedness for a digitally enabled health workforce.

## Introduction

The rapid development of artificial intelligence (AI) technologies is transforming numerous industries, including the field of education. The last few years have witnessed significant potential for AI to enhance the way knowledge is delivered, tested, and learned at every level of education, from common learning spaces to highly technical fields such as medical training. AI applications in medical education range from personalized learning systems, automated evaluations, and virtual simulated patients to complex decision-support systems that enable clinical thinking and learning for diagnosis [1-3]. These developments align with broader patterns across the health care industry, where innovations backed by AI technology are increasingly being integrated into clinical practice workflows and diagnostic systems [4,5].

Globally, several countries possessing robust health and education systems have actively incorporated AI into their medicine curricula. These cross-disciplinary programs embed AI competence and proficiency within undergraduate, postgraduate, and continuing education courses [6,7]. It is seen through scoping reviews that learning enriched by AI can potentially maximize learning efficiency, clinical preparedness, and adaptability to real-world medical problems [8,9]. A significant case is that of the Icahn School of Medicine at Mount Sinai, which in 2025 announced a full roll-out of ChatGPT Edu, a partnership with OpenAI for use in advancing education, research, and innovation for its programs [10]. All of this can be enabled only through robust infrastructure, long-term funding, and an innovative culture that supports transformational curricula and technology infusion.

In comparison, Vietnamese medical training is confronted by unique context-related issues. The pedagogical model remains strongly traditional, and hospital-based clinical attachments and didactic lectures dominate the learning environment. Limited digital infrastructure, uneven readiness among faculty members, and the shortage of national guidelines for integrating AI further limit innovation in teaching [11,12]. In addition, the disparity between AI development and pedagogical implementation is increasing across most low- and middle-income countries, and there are fears over learning inequity and readiness for the digital health workforce [13,14]. These inequalities explain the necessity to study the use, perception, and regulation of AI in Vietnamese medical education today.

Despite the significant focus on AI within international medical education, empirical research on preparedness, acceptance, and implementation of AI in the Vietnamese context is scarce. A study using a survey among pharmacy and medical students from the south region of Vietnam found that nearly all students had no idea what AI is and what it can do for health, while almost 80% thought it would be useful for their professional development [12]. There is a striking shortage when it comes to research on faculty members, institutional decision-makers, and policy-level interactions, but preparedness evaluations for AI within Vietnam’s health care system have identified issues ranging from limited sociopolitical support to inadequate information infrastructure [15]. This lack of data makes it difficult for evidence-based planning and for congruence between technological progress and reality within education (Textbox 1). In addition, although the global discourse on AI in medical education is expanding rapidly, Vietnam has received little scholarly attention despite its unique challenges of limited infrastructure, high student-to-faculty ratios, and uneven policy development. This viewpoint aims to address this gap by synthesizing existing literature on the use of AI in medical education, with a specific focus on the opportunities and challenges of integrating AI into Vietnamese medical education. This is intended for medical educators, institutional leaders, and policymakers who are navigating digital transformation in health professions training. We suggest that AI could play a key role in solving persistent issues such as overcrowded classrooms, limited clinical training opportunities, and unequal access to learning materials. However, several barriers stand in the way, including underdeveloped infrastructure, a general lack of digital and AI literacy, unclear ethical and legal frameworks, and hesitation around changing traditional teaching methods. Drawing on global developments, current research, and our own professional experience, we believe Vietnam needs a strategic, inclusive plan to integrate AI. This should focus on upgrading infrastructure, equipping both teachers and students with the necessary skills, reforming curricula, and establishing national-level guidelines. With the right approach, AI could not only bring Vietnamese medical education into the digital age but also help build a future-ready health workforce.

Textbox 1. **Gaps in the literature on artificial intelligence (AI) in medical education in Vietnam.**

Although artificial intelligence (AI) is gaining momentum in global medical education, current literature reveals critical gaps in the Vietnamese context that hinder evidence-based planning and risk misalignment between technological advances and educational realities.

**Lack of empirical research in medical education**

- Most existing studies on AI adoption focus on general higher education institutions rather than health professional training [11,13].

**Absence of national data on institutional readiness**

- There is limited insight into how medical schools and policy stakeholders perceive or plan for AI integration [15,16].

**Low AI literacy among health care students**

- A cross-sectional study found that 92.2% of students in southern Vietnam had no understanding of AI in health care, and 70.6% had never received formal instruction on the topic [12].

**Fragmented and small-scale initiatives**

- Although programs such as the Fulbright-Google collaboration [17] show promise, they are isolated efforts and are not embedded in national medical curricula.

**The gap between clinical AI use and educational application**

- While AI is being introduced in Vietnamese health care [14], there is a lack of studies on its application in teaching, assessment, or simulation-based learning in medical education.

## Characteristics of Medical Education in Vietnam

The Vietnamese system for training doctors has distinctive features at the structural and organizational level that are significantly different from high-income countries. There are about 30 universities across the country that offer undergraduate and postgraduate training for health sciences. A 6-year undergraduate program is typically completed by medical students to obtain a general medical doctor degree (MD, General Doctor).

Vietnamese teaching hospitals host, apart from undergraduate trainees, numerous other groups of learners simultaneously, including residents (Resident), master’s students (Postgraduate student, Master), and candidates for Specialty Level I and II (Specialist Level I Doctor, Specialist Level II Doctor), all of whom require clinical experience in the same health care facilities.

This diversity and volume of learners place significant pressure on the teaching infrastructure. Hospital clinical sites are often stretched not only by patient care demands but also by educational requirements. The student-to-patient ratio is generally low, which reduces opportunities for hands-on practice, case-based discussions, and guided clinical reasoning [18].

Moreover, many faculty members manage multiple roles, including classroom instruction, clinical supervision, and full-time medical duties, which results in fragmented teaching time and limits the capacity for educational innovation [11,12].

## Opportunities for AI in Vietnamese Medical Education

Lessons from global best practices highlight how AI can be adapted to resource-constrained settings similar to Vietnam. In Malaysia, pilot programs for AI literacy in medical schools have emphasized curriculum integration at the undergraduate level while addressing cultural resistance through faculty workshops [19]. A recent study conducted in Palestine reported high levels of AI adoption among medical students, with frequent use of tools such as ChatGPT (OpenAI) and virtual simulators. Students indicated that these technologies supported their academic performance and research productivity, particularly in tasks such as literature review and data analysis. Notably, students highlighted AI’s usefulness in improving time management by automating repetitive academic tasks, thereby enhancing overall efficiency [20]. A study from Oman reported that medical students demonstrated a moderate level of readiness to adopt AI as they transitioned into their clinical training years. ChatGPT emerged as the most frequently used AI tool, and notable differences in attitudes were observed between students with higher technological proficiency and those with limited digital skills. The authors emphasized that medical schools should integrate AI into their curricula to better prepare students for future clinical practice [21]. These experiences demonstrate the value of incremental implementation, regional collaboration, and leveraging domestic innovation ecosystems. For Vietnam, adapting such strategies could mean initiating AI pilots at a small number of universities, gradually expanding to provincial schools, and fostering partnerships with both local startups and global technology firms. This phased approach would ensure that AI adoption is context-sensitive, financially feasible, and culturally acceptable.

## Personalized Learning Experiences

AI facilitates the shift from 1-size-fits-all instruction to personalized teaching that adapts to the unique needs of each student. AI-powered adaptive learning systems continuously evaluate students’ progress using diagnostic tests and real-time performance monitoring. These systems can then dynamically adjust how content is delivered, providing remedial modules for those who need extra help with specific topics or accelerating the pace for advanced learners [22-24].

In Vietnam, where medical schools often struggle with high student-to-teacher ratios, such systems can play a vital role in improving educational equity. For example, though not yet widely adopted, AI-based platforms have demonstrated how they can deliver tailored feedback and interactive learning content for medical students [1]. In a recent survey of health care students in Vietnam, over 80% said they were willing to use AI tools to aid their studies, yet more than 90% had never actually used such technologies [12].

In addition, AI can be a valuable asset for students learning in English by offering translation, summarizing complex medical texts, or providing culturally relevant explanations. Chatbots such as ChatGPT or the Medical Pathways Language Model can act as around-the-clock learning companions, helping students understand difficult topics, review for exams, or practice clinical thinking through interactive questions-and-answers simulations [25].

## Enhanced Clinical Simulations

One of the most significant applications of AI in medical training is the use of clinical simulations combined with virtual reality, augmented reality, and natural language processing. These technologies can recreate high-stakes medical scenarios such as cardiac arrest, trauma response, or neonatal resuscitation, allowing students to make diagnostic and treatment decisions in a risk-free environment [26,27].

For example, AI-powered virtual patient platforms such as SimX (XR technology) and Body Interact (Take the Wind) offer students the opportunity to repeatedly practice, experience different case scenarios, and receive real-time feedback. These tools are designed to sharpen both psychomotor and cognitive skills, which are crucial for developing clinical competency [28,29]. In Vietnam, where access to real patient cases, particularly in rural training institutions, may be limited, such simulation-based learning can help fill crucial gaps in clinical exposure.

In addition, AI that uses the power of large language models (LLMs; advanced AI systems trained on vast amounts of text data, capable of generating human-like responses in natural language) can serve as a private coach to improve medical students’ soft skills. For example, the Artificial Intelligence Medical History Evaluation Instrument (the University of Arizona), an AI coaching tool developed by the Arizona Simulation Technology and Education Center, has been designed to enhance the communication skills of medical students. This system analyzes data from medical interviews conducted between students and patients, offering comprehensive evaluations of their interpersonal communication and medical competency skills. Assessment and feedback are provided according to guidelines from various organizations, including the Liaison Committee on Medical Education, the American College of Surgeons, and the World Health Organization. Owing to its automated nature, this technology provides significant opportunities for personalized coaching and can transform medical education in terms of time efficiency and cost-effectiveness. This technology may also be applicable in terms of scalability and broader access, which can be adapted to various medical education settings, including those in low- and middle-income countries.

AI also makes it possible to adjust the complexity of simulations based on a student’s performance. For instance, a student who successfully manages a simulated sepsis case might be presented with a more challenging case next time, one involving comorbid conditions or rare complications, following a mastery learning approach supported by AI [30].

## Efficient Administrative Processes

AI’s role in medical education goes beyond classroom instruction—it can also help resolve administrative challenges that are common in Vietnamese medical schools. For example, machine learning–based scheduling tools can streamline the creation of rotation timetables, classroom assignments, and faculty schedules. Software such as TimeTabler (October ReSolutions Limited) uses smart algorithms to handle logistical tasks that would otherwise demand extensive manual effort [31]. International experiences show that such systems can reduce scheduling conflicts dramatically and allow faculty to dedicate more time to teaching and mentoring.

AI also plays a role in assessment. It can be used to grade objective structured clinical examinations, multiple-choice questions (MCQs), and even written answers using natural language processing. Automated grading not only delivers faster feedback but also helps reduce human bias. Research shows that LLMs can support educators by generating standardized, objective structured clinical examination cases and evaluation rubrics. These AI tools can even benchmark student performance against expert evaluations [32,33]. In Vietnam, where faculty members often juggle teaching responsibilities across multiple institutions, such automation could reduce workload and improve consistency in student assessment.

Language processing capabilities allow AI tools to use LLMs to address issues related to medical education assessment. One particular domain that could significantly benefit from the application of LLM-based AI tools is the creation of MCQs for medical examinations. A multinational prospective study assessed the ability of ChatGPT to generate high-quality MCQs compared to university professors using standard medical textbooks [34]. The study showed that for the task of generating 50 MCQs for the graduate medical exam, ChatGPT took approximately 20 minutes, while professors took over 200 minutes. The analyses showed no significant differences in overall quality between MCQs generated by ChatGPT and by humans. This finding suggests that ChatGPT can produce MCQs of comparable quality to those generated by humans in a significantly shorter amount of time. It is important to note that since the publication of this study, more sophisticated LLM models with advanced reasoning capacity, including GPT-4 or GPT-5 (advanced LLMs developed by OpenAI), have been released by OpenAI, potentially outperforming the older models in such language processing tasks. Given that these models can be accessed via the application programming interface, tools that allow different software systems to communicate with each other—for example, enabling an educational platform to integrate AI functions—can automate this process without the need for manual input. Several tools using this approach have been developed, including Questgen (QuestgenAI Inc) or NoteGPT (NoteGPT AI Inc).

In Vietnam, where faculty members often juggle teaching responsibilities across various institutions or departments, automation like this can significantly lighten their load, giving them more time to focus on teaching and mentoring students.

In addition, predictive analytics powered by AI can help universities identify students who are at risk of failing based on their behavior and academic performance. This allows for timely intervention and support. Such tools are especially helpful in the competency-based education model that is gaining ground in Vietnamese medical training. International evidence supports the use of machine learning techniques such as k-nearest neighbors and exam performance predictors—to flag students who may need extra help [35,36]. In Vietnam, where competency-based education is being introduced, these systems could be integrated into student tracking to ensure timely support and reduce attrition. By combining automation with predictive tools, AI has the potential to not only ease administrative burdens but also strengthen educational outcomes through early intervention.

## Data-Driven Decision-Making

AI can analyze large sets of educational data to support quality improvements in medical education. For instance, when integrated into learning management systems, AI can monitor things such as student engagement, quiz scores, and participation in discussion forums. This type of data can help educators identify which parts of the curriculum are not working well, pinpointing specific modules or concepts that consistently challenge students [37]. In other countries, AI-powered dashboards have been used to redesign underperforming modules, improve exam blueprints, and assess the impact of new teaching methods, demonstrating clear benefits for curriculum quality assurance [38,39].

At the broader institutional level, AI can aid in accreditation and quality assurance by generating detailed dashboards that track metrics such as student learning outcomes, faculty performance, and the success of graduates. These insights can help academic leaders—such as deans and curriculum planners—make well-informed decisions about curriculum updates, faculty development, and student support systems [40,41].

In Vietnam, where a national competency-based medical education framework is in development, AI could play a crucial role. It can help map student progress against national competency standards and produce standardized performance reports across various medical schools [12].

An additional opportunity emerges from Vietnam’s health care digitization initiatives. With all hospitals nationwide mandated to implement electronic medical records by September 30, 2025 [42], medical schools will soon be connected to richer clinical data environments. This creates the possibility of linking students’ performance in clinical rotations with electronic medical records-based outcomes, allowing education to be evaluated alongside real-world patient care. Such integration could establish a feedback loop that improves both medical training and health care delivery.

Finally, AI-powered decision support systems can also assist policymakers by aggregating anonymized student and institutional data to forecast workforce needs. Predictive models could estimate shortages in specific specialties or geographic areas, ensuring that Vietnam’s medical education system aligns more closely with national health priorities. In a context where disparities in health care access remain significant, the use of data-driven decision-making could guide not only academic reform but also broader strategies for building an equitable health workforce.

## Challenges in Implementing AI in Vietnamese Medical Education

Table 1 summarizes the contrast between international adoption of AI in medical education and the Vietnamese context, highlighting the structural, educational, and policy gaps that shape the challenges discussed (Table 1).

**Table 1.** Comparison of artificial intelligence (AI) integration in medical education: international versus Vietnam.

Dimension	International context	Vietnamese context
Curriculum integration	Many universities embed AI^a^ literacy and competencies into undergraduate, postgraduate, and continuing medical education programs [43].	No national AI curriculum, minimal exposure, and most students report no formal training in AI in health care [12].
Infrastructure	Robust digital platforms, simulation centers, and cloud-based tools are widely available, with strong institutional investment [29,43].	Limited infrastructure, particularly in provincial schools and outdated labs, and poor internet continue to hinder AI adoption [11,16].
Faculty readiness	Faculty development programs in AI pedagogy and interdisciplinary teaching are increasingly common [6].	Faculty often lack AI knowledge, have limited training opportunities, and have a heavy clinical workload that further limits innovation [12,14].
Policy and regulation	Clearer data protection and AI ethics frameworks in regions such as the European Union and the United States [44].	A national AI strategy exists, but there are no specific laws for AI in education or student data protection.
Student exposure	Widespread use of AI tools for adaptive learning, automated assessment, and clinical simulation [1,22-25].	Over 90% of health care students have never used AI in their studies, despite high interest [12].
Cultural acceptance	Increasingly normalized as part of digital transformation in education [45].	Skepticism and resistance among faculty and uneven acceptance across institutions.

a AI: artificial intelligence.

## Limited Awareness and Understanding

A major obstacle to integrating AI effectively into Vietnamese medical education is the widespread lack of knowledge and basic understanding among both students and educators [12]. While discussions about AI in health care are growing globally, many medical trainees in Vietnam are still unfamiliar with its core concepts and practical applications. According to a 2023 cross-sectional study, 92.2% of health care students in southern Vietnam reported having no understanding of how AI works in health care, and 70.6% had never received any formal education on AI-related topics [12]. These figures highlight a significant educational gap and point to an urgent need to introduce AI literacy into the medical curriculum. The urgent call for this integration has also been sparked in Malaysia, a country located in the same geographic area as Vietnam, where unreadiness for AI has been reported widely among medical students [46].

Moreover, most medical schools in Vietnam still rely heavily on traditional, lecture-based teaching methods and have limited integration of digital tools. Without structured exposure to AI, whether through dedicated coursework, hands-on workshops, or interdisciplinary collaboration with departments such as data science, future health care professionals may lack the skills needed to use AI tools responsibly or interpret AI-generated clinical data. This lack of preparation not only hampers the adoption of AI in training but also raises long-term concerns about the quality of health care delivery as AI becomes increasingly embedded in global medical practice.

## Infrastructural Constraints

The effective integration of AI in medical education relies heavily on a strong technological foundation. This includes dependable high-speed internet, access to cloud computing, up-to-date hardware, and specialized software. Unfortunately, many medical schools in Vietnam, especially those in rural areas or linked to lower-tier universities, face significant infrastructure challenges. Common issues such as limited budgets, outdated computer labs, and a lack of technical support continue to obstruct the adoption of AI-powered learning tools [11,15,16].

As of now, no medical university in Vietnam has officially developed or launched an AI-based curriculum tailored to medical training. The digital divide between urban and rural institutions further exacerbates the issue, highlighting both a lack of preparedness and a deep educational disparity.

Globally, research shows that while AI is making strides in medical education in well-funded regions, many low- and middle-income countries lag due to insufficient infrastructure, a shortage of trained faculty, and limited institutional readiness [2,47]. These international gaps are mirrored within Vietnam, where centrally located universities may have the means and partnerships to explore AI, whereas many provincial medical schools simply lack the resources and expertise to do so.

Without unified national investment and clear policy direction to support digital transformation across all levels of medical education, AI advantages risk being confined to a few elite institutions, ultimately undermining efforts to build a fair and forward-looking health care workforce for the entire country.

## Quality Assurance, Ethical, Legal, and Policy Challenges

Ensuring the quality, validity, and reliability of AI-assisted assessment is critical in medical education, where evaluation outcomes have direct implications for patient safety and professional competency [48]. Although AI shows promise in automating grading and generating exam content, risks remain regarding algorithmic bias, variability, and transparency [49]. To safeguard assessment integrity, AI-generated items and evaluations should undergo external expert review and be pilot-tested before use in high-stakes examinations. Institutions should adopt national or regional standards for AI-assisted evaluation, ensuring consistency across medical schools. Moreover, regular audits of AI performance against human benchmarks are needed to detect errors or unintended biases. Establishing clear oversight mechanisms will be essential for building trust in AI-enhanced assessments and protecting both educational and professional standards [50].

The use of AI in education also brings forward a range of ethical challenges, especially around data privacy, informed consent, fairness in algorithms, and transparency. Many AI systems rely on collecting and analyzing extensive data such as student performance, learning habits, and, in some cases, biometric or voice information. AI-powered proctoring tools used during online exams can record facial movements, keystrokes, and background activity. While these tools are meant to prevent cheating, universities have faced student protests and lawsuits due to concerns about surveillance and data misuse. Without strong legal or institutional safeguards, there is a real risk of similar issues in Vietnam, where institutional data governance remains underdeveloped and student privacy could easily be compromised [51].

A significant concern is algorithmic bias. If an AI system is trained on unrepresentative data or lacks clear decision-making criteria, it may unfairly evaluate students. This is especially troubling in high-pressure fields such as medical education, where skewed assessments could influence a student’s confidence, academic progress, or even future career opportunities [2,52]. The lack of transparency in how these algorithms operate makes it even harder to hold them accountable.

As of 2024, Vietnam does not yet have a solid legal framework to govern the use of AI in higher education; it is important to situate this gap within the country’s broader digital transformation and health policy agenda. In 2020, the government launched the National Digital Transformation Program to 2025 with orientation to 2030, which identifies health care and education as 2 priority sectors for digital innovation [53]. The program calls for the adoption of digital platforms, cloud-based services, and data-driven management tools in universities and hospitals, laying a foundation that could facilitate the integration of AI into medical training.

Similarly, the Ministry of Health has advanced health care digitization initiatives under the “Smart Health” and National Health Digital Transformation plans [54], focusing on electronic medical records, telemedicine, and health information systems. According to recent regulations, all hospitals nationwide are mandated to implement electronic medical records by September 30, 2025 [42]. While these initiatives primarily target clinical service delivery, they create data ecosystems and digital infrastructure that could be leveraged for AI-driven medical education, especially in areas such as simulation, case-based training, and competency tracking.

Although early-stage policies have been suggested under the national AI development strategy, no specific laws are in place to address data protection, algorithmic responsibility, or the ethical role of AI in classrooms [16]. This gap raises pressing concerns about how institutions and regulators should manage these technologies responsibly.

Finally, while AI can enhance efficiency and scale learning, it risks sidelining the personal, human aspects of medical training. Skills such as empathy, ethical judgment, and effective communication are vital in health care, and they develop best through real human interaction and mentorship. AI may support education, but it cannot replace the essential human touch in shaping compassionate medical professionals [1,55].

## Cultural and Acceptance Issues

One of the key obstacles to integrating AI in Vietnam’s medical education system is the cultural and institutional resistance to change. Many faculty members and administrators, having been trained in conventional teaching methods, may approach AI with skepticism or even see it as a threat to their professional roles. Concerns about job security, diminished academic authority, or simply a lack of familiarity with emerging technologies can fuel this reluctance [56,57].

These attitudes are deeply rooted in Vietnamese pedagogical traditions, which remain strongly influenced by Confucian values emphasizing hierarchy, respect for authority, and teacher-centered learning. The traditional lecture-based approach positions the teacher as the primary source of knowledge, while students are expected to learn through memorization and obedience. In this context, the idea of AI systems providing personalized tutoring or automated feedback may be perceived as undermining the authority of faculty or disrupting established classroom dynamics.

Similarly, the relationship between faculty and students in Vietnamese medical schools often emphasizes formality and deference, which may affect how students engage with AI-driven tools. While younger generations may be more open to experimentation, students might hesitate to adopt AI-based tutoring systems if they sense disapproval from faculty or if doing so appears to challenge established norms of learning. Conversely, if faculty members actively endorse and model the use of AI, acceptance among students is likely to grow more rapidly.

Moreover, Vietnamese academic culture tends to value collective conformity over individual experimentation. Students often prioritize standardized examination performance, and learning strategies are adapted toward achieving success in high-stakes tests. Because AI tools often encourage exploratory, self-paced, and competency-based learning, there may be a mismatch between the innovative potential of AI and the exam-driven educational ethos. Unless AI is aligned with assessment systems and institutional incentives, it risks being perceived as peripheral or even irrelevant.

Addressing these cultural barriers requires meaningful investment in faculty development. Training programs that introduce educators to AI tools, illustrate their value in teaching, and offer hands-on learning opportunities can help build trust and competence. Promising signs of progress can be seen in local efforts such as Fulbright University’s AI education grant and VinBrain’s nationwide campaigns promoting AI in health care, both of which highlight the power of sustained institutional backing in shifting academic mindsets [17,58].

Leadership also plays a crucial role in fostering a culture of innovation. Universities need to actively support digital experimentation, offer incentives for early adopters, and ensure that AI initiatives align with broader goals such as competency-based education and health care system reform. Without genuine buy-in from faculty, there is a risk that AI will be viewed as a top-down mandate, poorly integrated into everyday teaching, and ultimately ineffective [2,24,59].

## Cost-Effectiveness and Sustainability

Cost-effectiveness and sustainability are central concerns for Vietnam as a middle-income country [60-62]. While AI technologies promise long-term benefits, including reduced faculty workload, standardized assessments, and improved student retention, their implementation requires upfront investment in infrastructure, training, and software. To ensure sustainability, policymakers and universities must consider the return on investment and adopt funding models that extend beyond 1-time grants. Public-private partnerships, collaborations with technology companies, and integration with national digital transformation and health care digitization programs represent potential pathways for sustainable financing [62]. Without deliberate strategies, there is a risk that only urban and better-resourced institutions will benefit, thereby deepening existing inequities with respect to provincial universities. Embedding cost-effectiveness evaluations into policy design and monitoring will be essential to ensure scalability and equitable distribution of AI benefits across the entire medical education system.

## Conclusion and Implications

Integrating AI into the Vietnamese medical education system offers a powerful opportunity to modernize how future health care professionals are trained. AI can individualize learning, simulate complex clinical scenarios, automate assessment, and guide responsive curriculum reform—advantages that are particularly relevant in Vietnam, where faculty shortages and unequal resource distribution persist—yet realizing these benefits requires more than enthusiasm. Low levels of AI literacy, uneven technological capacity, concerns about ethics and data protection, and resistance within academic culture remain significant barriers. The recommendations outlined in Textbox 2 provide a practical roadmap for short-, medium-, and long-term action. With deliberate planning, sustained investment, and national coordination, Vietnam can ensure that AI integration is not only feasible but also equitable and sustainable, ultimately strengthening the country’s preparedness for a digitally enabled health workforce.

Textbox 2. **Recommendations for the integration of artificial intelligence (AI) in Vietnamese medical education.**

**Short-term**

- Introduce artificial intelligence (AI) literacy modules in undergraduate and postgraduate curricula, covering:

-  Fundamentals of machine learning and large language models.
-  Principles of data ethics, bias awareness, and privacy.
-  Clinical applications of AI in diagnostics, simulation, and decision support.

- Require medical universities to conduct faculty development workshops on AI tools for teaching, simulation, and assessment.
- Pilot the use of AI-assisted exam generation and automated grading (eg, multiple-choice questions and objective structured clinical examination checklists) at selected universities.
- The responsible parties include individual universities, with oversight provided by the Ministry of Education and Training (MOET) and the Ministry of Health (MOH).

**Medium-term**

- Establish minimum infrastructure standards, including:

-  Reliable high-speed internet access.
-  Cloud-based learning management systems.
-  Secure institutional data storage and access to national health data platforms.

- Develop national ethical guidelines for AI in education, coordinated by MOET and MOH, with input from medical universities, professional associations, and legal experts.
- Expand pilot projects to provincial universities, accompanied by rigorous outcome evaluations.
- The responsible parties include MOET, MOH, national medical councils, and professional associations.

**Long-term**

- Integrate AI literacy as a core requirement across all medical training programs nationwide.
- Mandate external quality assurance mechanisms for AI-generated educational content and assessments to ensure validity and reliability.
- Create a sustainable funding framework through public-private partnerships and government-supported digital transformation budgets.
- Align AI-based educational analytics with national health workforce planning systems, ensuring that training outputs meet the needs of the health care system.
- The responsible parties include MOET, MOH, the Ministry of Science and Technology, and national accreditation bodies.

The integration of AI has broad implications for policy, practice, and research. Policymakers must establish clear ethical and regulatory frameworks and invest in robust digital infrastructure to prevent widening inequities between urban and provincial institutions. For educators and universities, AI provides practical solutions to faculty shortages, overcrowded classrooms, and limited clinical exposure, yet these benefits can only be realized if faculty development and support systems are prioritized. For researchers, there is an urgent need for Vietnam-specific studies evaluating the effectiveness, acceptability, and long-term outcomes of AI-based educational tools. Interdisciplinary collaborations between medical and technical institutions will be crucial in generating locally relevant evidence.

Drawing from the recommendations outlined in this paper, Vietnam should prioritize: (1) including AI literacy modules in undergraduate and postgraduate curricula, (2) investing in IT infrastructure and cloud-based educational platforms, particularly in provincial schools, (3) conducting faculty development programs focused on digital teaching tools, (4) establishing ethical guidelines for AI use in education to protect student data and ensure fairness, and (5) promoting interdisciplinary collaboration between medical and technology faculties.

By addressing these policy, practice, and research priorities, Vietnam can ensure that AI adoption not only modernizes medical education but also contributes to a resilient, equitable, and future-ready health workforce.

## Abbreviations

AI: artificial intelligence
LLM: large language model
MCQ: multiple-choice question
